# Predicting Effective Adaptation to Breast Cancer to Help Women BOUNCE Back: Protocol for a Multicenter Clinical Pilot Study

**DOI:** 10.2196/34564

**Published:** 2022-10-12

**Authors:** Greta Pettini, Virginia Sanchini, Ruth Pat-Horenczyk, Berta Sousa, Marianna Masiero, Chiara Marzorati, Viviana Enrica Galimberti, Elisabetta Munzone, Johanna Mattson, Leena Vehmanen, Meri Utriainen, Ilan Roziner, Raquel Lemos, Diana Frasquilho, Fatima Cardoso, Albino J Oliveira-Maia, Eleni Kolokotroni, Georgios Stamatakos, Riikka-Leena Leskelä, Ira Haavisto, Juha Salonen, Robert Richter, Evangelos Karademas, Paula Poikonen-Saksela, Ketti Mazzocco

**Affiliations:** 1 Applied Research Division for Cognitive and Psychological Science European Institute of Oncology IRCCS Milan Italy; 2 Department of Oncology and Hemato-oncology University of Milan Milan Italy; 3 Department of Public Health and Primary Care Centre for Biomedical Ethics and Law KU Leuven Leuven Belgium; 4 Paul Baerwald School of Social Work and Social Welfare The Hebrew University of Jerusalem Jerusalem Israel; 5 Breast Unit Champalimaud Clinical Centre/Champalimaud Foundation Lisboa Portugal; 6 Division of Breast Surgery European Institute of Oncology IRCCS Milan Italy; 7 Division of Medical Senology European Institute of Oncology IRCCS Milan Italy; 8 Department of Oncology Comprehensive Cancer Center Helsinki University Hospital and University of Helsinki Helsinki Finland; 9 Department of Communication Disorders The Sackler Faculty of Medicine Tel-Aviv University Tel-Aviv Israel; 10 Champalimaud Research and Clinical Centre Champalimaud Foundation Lisboa Portugal; 11 ISPA Instituto Universitário Lisboa Portugal; 12 NOVA Medical School NMS Universidade Nova de Lisboa Lisboa Portugal; 13 In Silico Oncology and In Silico Medicine Group Institute of Communication and Computer Systems Athens Greece; 14 School of Electrical and Computer Engineering Athens Greece; 15 National Technical University of Athens Athens Greece; 16 Nordic Healthcare Group Helsinki Finland; 17 Varian Medical System Helsinki Finland; 18 Department of Psychology University of Crete Crete Greece; 19 Foundation for Research and Technology - Hellas Heraklion Greece

**Keywords:** resilience, personality, coping, decision-making, cancer, quality of life

## Abstract

**Background:**

Despite the continued progress of medicine, dealing with breast cancer is becoming a major socioeconomic challenge, particularly due to its increasing incidence. The ability to better manage and adapt to the entire care process depends not only on the type of cancer but also on the patient’s sociodemographic and psychological characteristics as well as on the social environment in which a person lives and interacts. Therefore, it is important to understand which factors may contribute to successful adaptation to breast cancer. To our knowledge, no studies have been performed on the combination effect of multiple psychological, biological, and functional variables in predicting the patient’s ability to bounce back from a stressful life event, such as a breast cancer diagnosis. Here we describe the study protocol of a multicenter clinical study entitled “Predicting Effective Adaptation to Breast Cancer to Help Women to BOUNCE Back” or, in short, BOUNCE.

**Objective:**

The aim of the study is to build a quantitative mathematical model of factors associated with the capacity for optimal adjustment to cancer and to study resilience through the cancer continuum in a population of patients with breast cancer.

**Methods:**

A total of 660 women with breast cancer will be recruited from five European cancer centers in Italy, Finland, Israel, and Portugal. Biomedical and psychosocial variables will be collected using the Noona Healthcare platform. Psychosocial, sociodemographic, lifestyle, and clinical variables will be measured every 3 months, starting from presurgery assessment (ie, baseline) to 18 months after surgery. Temporal data mining, time-series prediction, sequence classification methods, clustering time-series data, and temporal association rules will be used to develop the predictive model.

**Results:**

The recruitment process stared in January 2019 and ended in November 2021. Preliminary results have been published in a scientific journal and are available for consultation on the BOUNCE project website. Data analysis and dissemination of the study results will be performed in 2022.

**Conclusions:**

This study will develop a predictive model that is able to describe individual resilience and identify different resilience trajectories along the care process. The results will allow the implementation of tailored interventions according to patients’ needs, supported by eHealth technologies.

**Trial Registration:**

ClinicalTrials.gov NCT05095675; https://clinicaltrials.gov/ct2/show/NCT05095675

**International Registered Report Identifier (IRRID):**

DERR1-10.2196/34564

## Introduction

### Overview

Breast cancer is responsible for 28% of all cancer cases in Europe, with more than 2 million new cases in 2018 [1]. Despite continued progress in this area of medicine, dealing with cancers, such as breast cancer, is becoming a major socioeconomic challenge, in part due to increasing incidence. Furthermore, mortality has decreased significantly, with the 5-year survival rate progressing from 75% to 90% for women with breast cancer [2], contributing toward very significant increases in the number of long-term survivors, but with potential long-term losses in quality of life (QOL). Thus, it is of crucial importance to understand which psychological, social, contextual, and physical factors may affect or boost successful adaptation to breast cancer and its treatment. Accruing evidence [3,4] has defined the process of successful adaptation to chronic diseases, such as breast cancer, as “resilience.” Resilience is a complex and multidimensional construct that can be defined at different levels: as the individual’s potential (ie, the capacity to engage in adaptive coping processes), as a process (ie, the adaptive reaction to adversity), and as an outcome (ie, the final state achieved as the result of coping). A significant effort to reach a consensus definition was made by Southwick and colleagues [4], according to whom resilience includes “healthy, adaptive, or integrated positive functioning over the passage of time in the aftermath of adversity.” This definition highlights the two main components of resilience: the presence of adversity and the positive adaptation to it [5]. In fact, when faced with potentially life-threatening events, each person engages in coping strategies that can vary widely in the capacity to provide adaptive solutions and to ensure optimal recovery with respect to the disease itself, as well as to overall QOL.

Important questions remain regarding the determinants of resilience and how it can be measured. Consensus exists that it should be analyzed with a multilevel perspective, including biological, demographic, cultural, economic, psychological, behavioral, and social variables [4]. As such, interest in the impact of biological factors on resilience has increased, with several studies, including research in animal models investigating processes akin to resilience, having shown an association between resilience, inflammation, and immune processes, similar to pathways that have been described in aging [6] and cancer [7-11]. However, there is also evidence that other factors, such as sociodemographic [12-14] and psychological characteristics as well as social environment [15], impact the ability of people with individual differences to manage and adapt to the entire cancer care process. Here we describe the rationale and methods for a study to assess resilience multidimensionally in women with breast cancer.

### Sociodemographic and Psychological Characteristics in Cancer Adjustment

Over the last few decades, interest in the contribution of patient characteristics on cancer onset, treatment, and management as well as the ability to cope with cancer have widely increased [16-18]. Higher levels of resilience have been described in patients of younger age, female sex, and higher socioeconomic status as well as those who are married [12-14]. In addition to sociodemographic characteristics, other internal (eg, personality traits, dispositional optimism, and self-efficacy) and external (eg, social support) factors may affect the resilience of patients with cancer [15]. For example, adopting cognitive regulation strategies may help patients cope with strong emotions in order to not get overwhelmed and avoid stressful outcomes [19,20]. In line with these findings, acceptance attitudes and positive thinking also seem to play an important role in patients’ psychological well-being, while rumination and catastrophizing often lead to negative emotions [21-25]. Ultimately, high resilience levels could affect adherence to treatment procedures, thus promoting faster recovery and lower clinical burden [15].

Regarding patients with breast cancer, personality traits may significantly affect psychological status in the process of adaptation to the disease. A recent study showed dispositional optimism as an important short- and long-term predictor of psychological well-being after breast cancer, where patients with more optimistic orientation reported lower distress levels, whereas unpleasant emotions were mainly experienced by people with a pessimistic approach [26]. Furthermore, self-efficacy appears to be associated with higher levels of wellness, better QOL, and decreasing depression and anxiety, even 1 year after diagnosis [27]. In addition, perceived social support acts as a protective factor, allowing better adaptation and promoting positive coping strategies in patients with breast cancer [28]. In line with these findings, it is known that a cancer diagnosis affects not only the patient but also his or her family system, which represents a key source of support for better adaptation to the disease [29]. As an example, in 2018, Faccio and colleagues [30] proposed a model of family resilience that highlighted the key role of family in the patient’s decisions and overall well-being. In fact, a cancer diagnosis can be considered as a perturbation of the whole family system, which may result in a smooth adaptation to a new homeostasis or in difficulties that prevent the readjustment process [31]. According to this model, higher cohesion and more clear and consistent communication among the family members, in addition to the possibility of sharing feelings and fears, increase the patient’s ability to organize her experience and adapt to the new condition [32].

### Novelty and Study Aim

While several theoretical contributions regarding resilience in medical settings have already been published [33], to our best knowledge, no studies have been performed on the combined effect of multiple psychological, biological, and functional variables in predicting the ability of patients to bounce back from a stressful life event, such as a breast cancer diagnosis. There is a growing need for novel strategies to improve the capacity to predict resilience in response to a variety of stressful experiences, including breast cancer. A major objective for the field is to enhance resilience in the face of breast cancer, and its prediction would, thus, be a necessary step toward efficient recovery through personalized interventions.

The bidirectional relationship between medical and psychosocial factors in breast cancer has been well established in previous studies. For example, breast cancer treatments, such as radiation and chemotherapy, have been associated with higher levels of psychological distress, long-term cognitive dysfunction, and lower QOL [34-36]. Consistently, several studies have estimated the prevalence of depression in the early stages of breast cancer to be around 15% to 20% [36,37], whereas Burgess and colleagues [38] found that almost 50% of women with breast cancer report anxiety or depression symptoms in the first year after diagnosis. The prevalence of these symptoms drop to 25% in the second year but remain as high as 15% thereafter [38]. There is evidence that several psychological factors affect the progress of disease. Higher psychological distress, for example, may lead to additional medical examinations, may negatively affect treatment decision-making, and could even disrupt ongoing medical treatments [39,40]. There is also evidence that psychosocial factors, including distress, stressors, low optimism, and poor social support, have an impact on immune responses (eg, lymphocyte proliferation), on physiological activation (eg, along the hypothalamic-pituitary-adrenal axis), and on lifestyle behaviors (eg, smoking and medication adherence), consequently affecting the course of disease [39,41-43].

**Figure 1:**
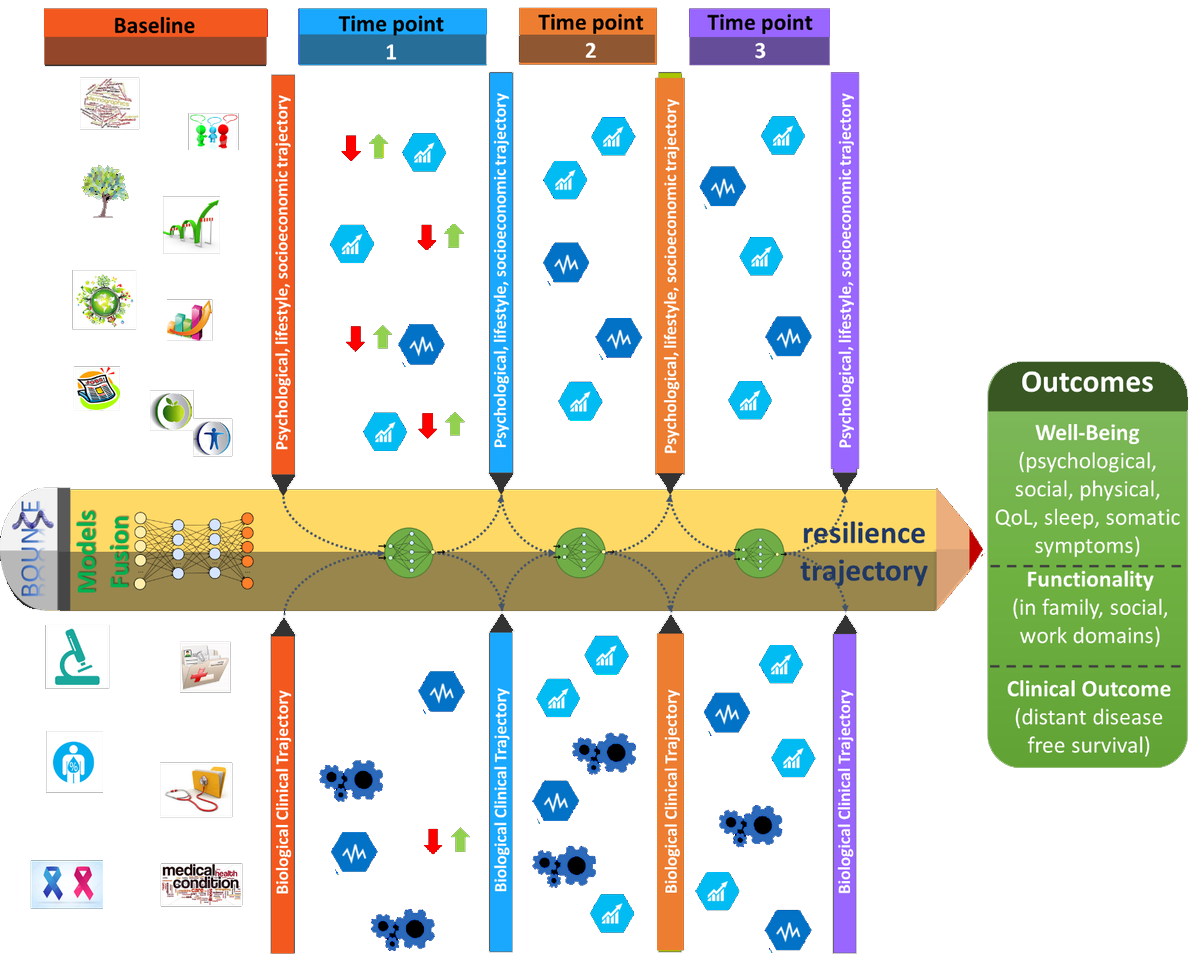
Schematic illustration of the multiple factors affecting the resilience trajectory and outcomes. QoL: quality of life (adapted from Poikonen-Saksela [44], with permission from Paula Poikonen-Saksela).

Drawing from the theoretical and empirical framework described above, here we describe the study protocol of a multicenter clinical study entitled “Predicting Effective Adaptation to Breast Cancer to Help Women to BOUNCE Back” or, in short, BOUNCE. With this study, we intend to identify psychosocial, biomedical, and functional factors that predict the capacity of individual patients to “bounce back” during the highly stressful treatment and recovery period following a diagnosis of breast cancer (Figure 1 [44]). This study has been designed to investigate resilience trajectories starting at diagnosis and for 18 months of follow-up. The underlying hypothesis is that biomedical, psychosocial, and functional factors may predict trajectories of resilience and adjustment to breast cancer.

If confirmed, this would support the general purpose of the study, which is the early identification of women at risk for whom early intervention (eg, through personalized psychological support) would be necessary. This multicenter clinical pilot study is the core of a larger European Union (EU) project named BOUNCE (grant agreement No. 777167), which has been developed to understand and study resilience through the cancer continuum in patients with breast cancer. The main global goal of the BOUNCE project is to build a quantitative mathematical model of factors associated with the capacity for optimal adjustment to cancer; this will initially be done through a data-driven method, including the computation of resilience on the basis of retrospective data, and through a psychometric method, as described here, with prospective assessment of several domains of resilience through questionnaires.

### Objectives

The primary objective of this multicenter clinical pilot study is to identify the interplay of clinical, biomedical, and psychosocial factors in predicting patients’ resilience to breast cancer at several time points after diagnosis.

The secondary objectives are as follows:

1. Differentiating between trajectories of psychological adaptation to breast cancer.
2. Developing a multidimensional index of resilience as a function of biomedical status, psychosocial status, and functional status.
3. Examining potential differences in the predictive and outcome variables across the four clinical sites in which the study is conducted.
4. Cross-validating the prediction models in order to assess the accuracy of their performance in practice and to enhance their generalization.

## Methods

### Study Design and Clinical Partners

The clinical pilot study has been designed as a longitudinal prospective cohort, with assessments at diagnosis of breast cancer and 3, 6, 9, 12, 15, and 18 months later. It involves different clinical centers in several countries, namely the European Institute of Oncology (IEO) in Italy, Helsinki University Hospital (HUS) in Finland, the Champalimaud Clinical Centre (CHAMP) in Portugal, and the Rabin and Shaare Zedek Medical Centers, coordinated by the Hebrew University of Jerusalem (HUJI) in Israel. Data were primarily collected through the Noona Healthcare platform (Noona), a personal health records system designed for patients with cancer. For those patients who do not want or are unable to use Noona, the paper-and-pencil mode are available. Data are then inserted into the Noona platform by a researcher. Noona is a fully responsive web application that is usable with a web browser on any suitable device that is available to the user, including desktop, laptop, tablet, and smartphone devices. Noona is classified as a medical device; more specifically, it is classified as a web platform designed for patients with cancer for remote monitoring and as a support tool for communication between patients with cancer and health care professionals. However, in the multicenter clinical study, Noona will not be used for communication between the treatment team and patients, but will only be used for the collection of study-relevant information. Thus, the main functionalities, such as reporting symptoms or requesting assistance, will not be taken into consideration. The study was registered at ClinicalTrials.gov (NCT05095675).

### Study Participants

Women with histologically confirmed stage I to III breast cancer have been recruited across the several study centers, with 660 patients recruited at baseline. Details on the inclusion and exclusion criteria are provided in the Selection Criteria section below.

### Selection Criteria

Inclusion criteria include the following:

- Female patients, 40 to 70 years of age at the time of diagnosis
- Histologically confirmed invasive breast cancer, early or locally advanced but operable
- Tumor stage I, II, and III
- Patients receiving surgery as part of the local treatment
- Patients receiving systemic treatment for breast cancer, regardless of treatment type
- Informed consent form signed.

Exclusion criteria include the following:

- Refusal to provide informed consent
- Presence of distant metastases
- History of another malignancy or contralateral invasive breast cancer within the last 5 years, with the exception of cured basal cell carcinoma of skin or carcinoma in situ of the uterine cervix; the patient’s resilience could have been affected by a previous cancer diagnosis
- History of an early-onset (ie, before 40 years of age) mental disorder (eg schizophrenia, psychosis, bipolar disorder, and diagnosis of major depression) or severe neurologic disorder (ie, a neurodegenerative disorder and dementia)
- Other concomitant serious diseases that could affect a patient’s resilience and cancer pathway, such as clinically significant (ie, active) cardiac disease (eg, congestive heart failure, symptomatic coronary artery disease, or cardiac arrhythmia not well controlled with medication) or myocardial infarction within the last 12 months
- Major surgery for severe disease or trauma that could affect a patient’s psychosocial well-being (eg, major heart or abdominal surgery) within 4 weeks before study entry, or lack of complete recovery from the effects of surgery
- Treatment for other invasive cancer
- Treatment for any major illness in the last 6 months
- Pregnancy or breastfeeding at the time of recruitment.

### Instruments and Measures

#### Psychosocial Instruments

The processes of defining the instruments started with a list of 50 relevant psychological constructs and their measures that were hypothesized to affect resilience. This initial pool was determined in accordance with the results of literature research and the research experience of each of the four clinical teams. The following criteria were used to define instruments for data collection: (1) sound psychometric properties (ie, reliability and construct validity), (2) divergent validity in the context of this research (ie, low overlap with other measures), (3) ability to predict important outcomes in the cancer resilience trajectory or in longitudinal studies (ie, controlling for initial levels of the outcome measures), and (4) reduced number of items. The final questionnaire consists of a set of validated measurement tools related to the following domains: personality, meaning, comprehensibility and manageability of the disease, trauma exposure, coping, social support, resilience, illness perception, QOL, and distress. The scales used to measure such domains are reported in Table 1 [45-63].

The collection of sociodemographic and lifestyle variables includes information about age, level of education, marital status, number of children, employment status and sick days, flexible arrangements at work, return to work, income, faith, smoking and alcohol consumption, drug use, weight and height, diet, exercise, number of professional support sessions, variations in family’s work, other leisure activities, and presence of domestic help (Table 2).

**Table 1:** Psychosocial assessment tools.

Domain and measure names		Month^a^						
		0	3	6	9	12	15	18
**Personality**								
	Ten-Item Personality Inventory [45]	✓						
	Life Orientation Test–Revised [46]	✓						
**Meaning**								
	Sense of Coherence scale [47]	✓						
**Trauma exposure**								
	PTSD^b^ Checklist [48]			✓		✓		✓
	Recent negative life events	✓	✓	✓	✓	✓	✓	✓
	Recent illness		✓	✓	✓	✓	✓	✓
	Posttraumatic Growth Inventory [58]		✓			✓		✓
**Coping**								
	Perceived Ability to Cope with Trauma scale [49]	✓			✓		✓	
	Cognitive Emotion Regulation Questionnaire [50]	✓			✓		✓	
	Mindful Attention Awareness Scale [51]	✓				✓		
	Mini–Mental Adjustment to Cancer Scale [56]		✓		✓		✓	
	Single item: What have you done to cope?		✓	✓	✓	✓	✓	✓
	Spirituality coping—a visual analog scale		✓		✓		✓	
**Social support**								
	Modified Medical Outcomes Study Social Support Survey [52]		✓		✓		✓	
	Family Resilience Questionnaire [32]		✓		✓		✓	
	Instrumental and emotional perceived social support	✓						
**Resilience**								
	Connor-Davidson Resilience Scale [53]	✓			✓		✓	
	Single item: How much are you back to yourself?			✓	✓	✓	✓	✓
**Illness perception and behaviors**								
	Illness Perception Questionnaire [54]			✓		✓		✓
	Items 3 and 4 from the Brief Illness Perception Questionnaire [55]		✓	✓	✓	✓	✓	✓
	Cancer Behavior Inventory [57]	✓		✓		✓		
	Modified Medical Outcomes Study Social Support Survey [52]		✓	✓	✓	✓	✓	✓
**Quality of life**								
	EORTC^c^ Quality of Life Questionnaire [59]	✓	✓	✓	✓	✓	✓	✓
	EORTC Quality of Life Questionnaire breast cancer module [59]	✓	✓	✓	✓	✓	✓	✓
**Distress**								
	Fear of Cancer Recurrence Inventory–short form [60]	✓		✓		✓		✓
	Hospital Anxiety and Depression Scale [61]	✓	✓	✓	✓	✓	✓	✓
	Positive and Negative Affect Schedule–short form [62]	✓	✓	✓	✓	✓	✓	✓
	Distress Thermometer [63]	✓	✓	✓	✓	✓	✓	✓

^a^A checkmark indicates that the assessment tool was administered at the indicated time point.

^b^PTSD: posttraumatic stress disorder.

^c^EORTC: European Organisation for Research and Treatment of Cancer.

**Table 2:** Sociodemographic and lifestyle assessments.

Variables	Month^a^						
	0	3	6	9	12	15	18
Year of birth	✓						
Level of education	✓						✓
Marital status	✓						✓
Number of children	✓						✓
Employment status	✓	✓	✓	✓	✓	✓	✓
Monthly income	✓						✓
Sick leave days	✓	✓	✓	✓	✓	✓	✓
Employer’s support					✓		✓
Return to work							✓
Level of religious faith	✓						✓
Smoke	✓			✓			✓
Drinking habits	✓			✓			✓
Use of drugs	✓		✓		✓		✓
Weight	✓		✓		✓		✓
Height	✓		✓		✓		✓
Diet	✓			✓			✓
Physical exercise	✓		✓		✓		✓
Mental health support		✓	✓	✓	✓	✓	✓
Support activities		✓	✓	✓	✓	✓	✓
Domestic help		✓	✓	✓	✓	✓	✓
Instrumental family support		✓	✓	✓	✓	✓	✓

^a^A checkmark indicates that the information was collected at the indicated time point.

#### Medical and Treatment Information

The clinical variables, including medical and treatment data, were retrieved from each patient’s health record. In particular, the following clinical variables were collected for each participant: classification by the International Statistical Classification of Diseases and Related Health Problems, 10th Revision; tumor biology (ie, primary tumor, regional lymph nodes, histological type, grade, estrogen receptor, progesterone receptor, and human epidermal growth factor receptor 2 [HER2]); surgery type and side; performance status; ongoing oncological therapy (ie, chemotherapy, endocrine therapy, anti-HER2 therapy, and radiotherapy); menopausal status; genetic risk factors; psychotropic medication; and comorbidity and laboratory tests, including hemoglobin, leukocytes, thrombocytes, neutrophils, and high-sensitivity C-reactive protein (Table 3). Furthermore, data on the patient care pathway were collected. These data were related to three different contexts: oncologic clinic, specialized care unit, and primary care or occupational health care. In particular, we were interested in collecting information regarding the following: the number of consultations with oncologists, nurses, psychiatrists, psychologists, and other health care professionals; the number and dates of treatment visits; the number and dates of inpatient days; the number of visits with regard to emergency care, laboratory visits, and imaging visits; and a list of prescribed medication. Finally, additional medical information was collected at months 12 and 18, regarding local relapse, metastatic disease, and death (Table 3).

**Table 3:** Medical assessment.

Variables		Month^a^						
		0	3	6	9	12	15	18
**Medical information**								
	Cancer stage	✓						
	Comorbidity	✓						
	Genetic risk factor	✓						
	Menopausal status	✓				✓		
	Tumor pathology	✓						
	Eastern Cooperative Oncology Group score	✓	✓	✓		✓		
	Psychotropic medication	✓	✓	✓		✓		
	Hormone replacement treatment	✓						
	Laboratory tests	✓				✓		
**Treatment information**								
	Surgery			✓				
	Chemotherapy			✓				
	Endocrine therapy					✓		✓
	Anti–human epidermal growth factor receptor 2					✓		✓
	Radiotherapy			✓				
	Side effects					✓		
Patient care pathway data			✓	✓	✓	✓		✓

^a^A checkmark indicates that the information was collected at the indicated time point.

### Time Point Measurements

Psychosocial, sociodemographic, lifestyle, and clinical variables were measured at different time points (Tables 1-3). There were seven assessment time points over a period of 18 months: baseline (ie, just after the diagnosis, before the start of chemotherapy, or within 2 weeks from the start of endocrine therapy) and every 3 months until month 18. Each assessment time point contains a set of specific measures that are able to capture salient changes in specific domains. Accordingly, variables that are not sensitive to change (eg, tumor biology and personality trait) were collected only at baseline. Data that are expected to change over time because of intervening factors (eg, starting of treatment and associated side effects, as well as adverse life events) were collected periodically.

### Recruitment and Follow-Up

A trained researcher identified all eligible patients, evaluating inclusion and exclusion criteria, by checking the patient’s health records stored in the electronic database of the hospital. Successively, during the first clinical consultation, the investigator briefly introduced the study to each eligible patient and collected informed consent from those interested in participating. During this first meeting, the investigator gave a short training in Noona and created the patient account. The investigator could use Noona to monitor the response status of questionnaires for each patient and could contact them to stimulate adherence to the study, completion of the questionnaires, and support for any issues with the platform.

### Statistical Procedures

#### Statistical Considerations on the Design

Data collected by Noona are stored centrally by the Foundation for Research and Technology–Hellas (FORTH); the data will be cleaned, homogenized, and shared with the Institute of Communications and Computer Systems (ICCS) for joint conduction of analyses. Interim analyses and quality checks will be conducted on data extracted at different time points during the project (eg, after the month 6 data are complete). Descriptive statistics (ie, mean, SD, median, maximum and minimum, and graphical representation) will be used to summarize the continuous data. Discrete measures will be summarized using counts, percentages, and graphical representations. Bivariate charts will be produced whenever desired. Temporal data mining, time-series prediction, sequence classification methods, clustering time-series data, and temporal association rules will be used to develop and validate the predictive model. Mediation, moderation, and moderated mediation analyses have a central role in the statistical methodology. In order to investigate whether the modality of data collection (ie, paper and pencil or the eHealth platform, Noona) will affect results on self-reported outcomes, analysis will be performed stratifying patients based on the method used to respond. Similarly, patients will be stratified based on sociodemographic (eg, country) and clinical variables (eg, cancer stage).

#### Sample Size Considerations

In the context of conventional statistical approaches, such as multiple linear regression, that have typically been employed in the existing relevant literature, a minimal sample size (n=500, considering maximal attrition rates of approximately 40%) is sufficient to ensure 85% power at *P*<.05; this will allow detection of the cumulative contribution of up to 30 independent predictors accounting for as little as 5.3% of total variance of each key study outcome. Furthermore, in a regression model with 30 independent variables, this sample size is sufficient to detect the significant added value of each independent variable, assuming a small effect size (Cohen *f*^2^>0.018). It should be noted, however, that this study proposed, for the first time, the use of nonconventional computational approaches to assess the hypothesized predictive variables. Given the complexity of the data sets and the fact that interactions between parameters are often difficult to specify—a requirement of conventional methods—supervised machine learning methods are emerging as the approach of choice for identifying hidden patterns among predictor variables. Perhaps the most distinct advantage of these methods is their adaptive capacity (ie, their inherent ability to optimize parameter weights based on known individual outcomes). The clinical accuracy of each optimized prediction model will be tested through various cross-validation techniques. 

### Data Collection Storage and Security

The multicenter pilot study involves the collection of personal data. Therefore, issues regarding confidentiality, privacy, and protection of data have been addressed so as to be compliant with Regulation (EU) 2016/679 of the European Parliament and of the Council of 27 April 2016 on the protection of natural persons with regard to the processing of personal data and on the free movement of such data, and repealing Directive 95/46/EC (General Data Protection Regulation). All data collected through the questionnaires and all relevant information about participants are stored in Noona electronic databases. The data are processed using a coding system that allows for the identification of patient identity only if and when necessary for the scientific objectives of the research project.

The lists below reflect the partners involved in the project and their respective roles regarding data collection, storage, and security.

Clinical partners are as follows:

- IEO: promoter of the multicenter pilot study and data controller
- HUS: coordinator of the EU BOUNCE project and data processor
- Rabin Medical Center and the Shaare Zedek Medical Center, under HUJI: data processor
- CHAMP: data processor.

Technical partners are as follows:

- FORTH: data analysis and storage
- ICCS: data analysis
- SingularLogic: data analysis and model development
- Nordic Healthcare Group: model development
- Noona: data storage.

### Ethics Compliance

Since the IEO is the promoter of the multicenter pilot study, the research protocol of the multicenter clinical study was first submitted for approval to the Ethics Committee of the IEO. Once approved by this Ethics Committee (approval No. R868/18-IEO 916; approval date: October 24, 2018), the protocol was submitted for approval to the Ethics Committees of HUS, the Rabin Medical Center and the Shaare Zedek Medical Center (under HUJI’s responsibility), and the CHAMP. In the course of the study, an amendment was submitted to the Ethics Committee of the IEO (amendment version No. 1 date: August 12, 2019; approval date: September 18, 2019) and to the centers in which such process was deemed necessary.

The multicenter pilot study has been devised so as to comply with both national (ie, Good Clinical Practices) and international declarations (ie, the Declaration of Helsinki) regulating proper ethical research involving human subjects, with informed consent obtained from all subjects. Specifically, conduction of the trial is in accordance with the following regulation and norms:

- The Declaration of Helsinki, ethical principles for medical research involving human subjects, revised October 2013
- The Convention for the Protection of Human Rights and Dignity of the Human Being with regard to the Application of Biology and Medicine: Convention on Human Rights and Biomedicine, Oviedo 1997
- The Council for International Organizations of Medical Sciences in collaboration with the World Health Organization, International Ethical Guidelines for Biomedical Research Involving Human Subjects, revised in 2016
- The Belmont Report: Ethical Principles and Guidelines for the Protection of Human Subjects of Biomedical and Behavioral Research. Department of Health, Education, and Welfare (DHEW) publication (DHEW-05-78-0012), Washington, DC, 1978.

## Results

The recruitment process stared in January 2019 and ended in November 2021. Preliminary results have been published in a scientific journal and are available for consultation on the BOUNCE project website [64]. Data analysis and dissemination of the study results will be performed in 2022.

## Discussion

Due to the increasing interest in the role of resilience in cancer recovery, there is a need for evidence regarding the complex paths between resilience, factors affecting resilience, and the interrelated illness outcomes. The study described here has the advantage of firstly assessing the combination of biological, clinical, lifestyle-related, and cognitive-emotional factors. These multiple variables account for systematic fluctuations of resilience across time that, in turn, actually contribute to successful adaptation to and recovery from breast cancer. BOUNCE aspires to move from a biomedical to a person-centered and biopsychosocial approach, toward growing awareness of the complexity of health and individual responses to illness [64-68]. In particular, we expect that this study will provide a predictive model to describe individual resilience and related factors, with the aim of personalized treatment plans, according to such predictions. In fact, we propose that the identification of individual resilience trajectories along the care process will lead to the development and implementation of tailored interventions, ideally supported by eHealth technologies [69-72]. In addition, it will scale up the knowledge about the interaction between biological, clinical, and psychosocial factors and outcomes, supporting, for example, programs of psychological prevention to support the patient across the disease trajectory.

Data collected from this pilot study could also provide information about cross-cultural differences. Further analyses and research based on BOUNCE data could examine the impact that the various health care systems in different countries could have on breast cancer resilience trajectories. Such possible differences will be of paramount importance, not only for a mere descriptive purpose but also for the development of a culture-based prediction model.

A possible limitation of this study is the large number of self-report scales used to collect the psychosocial variables; this may be perceived as too taxing by patients. However, considering that the general purpose of the study is the early identification of women at risk of poor adaptation, throughout the development of a data-driven quantitative mathematical model, all mentioned variables are needed at this research step to build a unified, multidimensional resilience trajectory predictor tool. This prediction model will encompass the results of the major analyses to be performed within the BOUNCE project; the results will identify potentially different trajectories of psychological adaptation to breast cancer over time, as well as the medical, sociodemographic, and psychosocial variables that may predict these trajectories. Although the final form of this tool is not yet decided and will depend on the actual findings of the study, hopefully it will allow health professionals to have a holistic picture of the patient condition: all the biological, psychological, and social factors together at a glance for a perfectly integrated treatment plan.

## Abbreviations

CHAMP: Champalimaud Clinical Centre
DHEW: Department of’ Health, Education, and Welfare
EU: European Union
EudraCT: European Union Drug Regulating Authorities Clinical Trials Database
FORTH: Foundation for Research and Technology–Hellas
HER2: human epidermal growth factor receptor 2
HUJI: Hebrew University of Jerusalem
HUS: Helsinki University Hospital
ICCS: Institute of Communications and Computer Systems
IEO: European Institute of Oncology
Noona: Noona Healthcare platform
QOL: quality of life
